# A Novel Hybrid Firefly Algorithm for Global Optimization

**DOI:** 10.1371/journal.pone.0163230

**Published:** 2016-09-29

**Authors:** Lina Zhang, Liqiang Liu, Xin-She Yang, Yuntao Dai

**Affiliations:** 1 College of Automation, Harbin Engineering University, Harbin, China; 2 School of Science and Technology, Middlesex University, London, United Kingdom; 3 College of Science, Harbin Engineering University, Harbin, China; Beihang University, CHINA

## Abstract

Global optimization is challenging to solve due to its nonlinearity and multimodality. Traditional algorithms such as the gradient-based methods often struggle to deal with such problems and one of the current trends is to use metaheuristic algorithms. In this paper, a novel hybrid population-based global optimization algorithm, called hybrid firefly algorithm (HFA), is proposed by combining the advantages of both the firefly algorithm (FA) and differential evolution (DE). FA and DE are executed in parallel to promote information sharing among the population and thus enhance searching efficiency. In order to evaluate the performance and efficiency of the proposed algorithm, a diverse set of selected benchmark functions are employed and these functions fall into two groups: unimodal and multimodal. The experimental results show better performance of the proposed algorithm compared to the original version of the firefly algorithm (FA), differential evolution (DE) and particle swarm optimization (PSO) in the sense of avoiding local minima and increasing the convergence rate.

## Introduction

Global optimization is crucially important in many applications, such as image processing [1], antenna design [2], chemistry [3], wireless sensor network [4], and so on. However, such global optimization problems are challenging to solve because these problems are often highly nonlinear with multiple local optima. Thus, traditional methods such as the gradient-based methods usually struggle to deal with such problems. Thus, for decades, researchers have attempted many different ways to try to solve such challenging problems with different degrees of success. In recent years, many researchers have proposed some new optimization algorithms [5–7].

Technically speaking, optimization methods can be divided into two main parts: deterministic algorithms and stochastic algorithms [8]. Deterministic algorithms such as the Hill-Climbing [9], Newton-Raphson [10] and Simplex Method [11] can get the same final results if the same set of initial values are used at the beginning. The advantages of such deterministic algorithms are that they usually have good efficiency for certain problems and require only a small number of iterations. However, one of their main disadvantages is the high probability of being trapped in local optima because they are local search algorithms. On the other hand, stochastic algorithms often use some randomness in their strategies which can enable the algorithm to escape from the local optima to search more regions on a global scale. This kind of strategy always produce unrepeatable routes of each individual run even starting with the same initial points. Though may be slightly different, the final results of these algorithms can often converge to the same optimal results within a given criterion if the algorithm is allowed to run long enough [8].

Nowadays, most stochastic algorithms can be called meta-heuristic algorithms [12]. Most of them have been developed, based on the biological processes in nature and these algorithms start to show their power and efficiency. Genetic Algorithm (GA) [13], Ant Colony Optimization (ACO) [14], Particle Swarm Optimization (PSO) [15–18], Artificial Bee Colony (ABC) [19], Cuckoo Search (CS) [20] and Firefly Algorithm (FA) [21–24] are some of the most popular algorithms in this class of stochastic algorithms. The disadvantages of these algorithms are the need for proper setting the algorithm-dependent parameters and a large number of iterations. However, these meta-heuristic algorithms have two main advantages. One is the good information-sharing mechanism which can promote the algorithm to converge faster under certain conditions and the other is the lower probability of entrapment into local modes.

The paper is organized as follows: the main idea of the standard firefly algorithm and standard differential evolution are illustrated in Section 2, and then the details of our proposed hybrid firefly algorithm are described in Section 3. In Section 4, we will demonstrate and carry out the analysis of the experimental results. Finally, Section 5 concludes the work.

## Firefly Algorithm and Differential Evolution

Firefly algorithm (FA) [25] is a new biologically inspired meta-heuristic optimization algorithm, which was proposed by Xin-She Yang in 2008. This algorithm is inspired by the flashing behaviour of tropical fireflies. Differential evolution (DE) [26] developed by Storn and Price in 1997 is also a meta-heuristic algorithm. DE with a potential parallel structure is a non-gradient-based, evolutionary computation algorithm. It has been proven that both algorithms can get a better optimal results than those achieved by the existing methods.

### Standard Firefly Algorithm

The Firefly Algorithm (FA) is based on the communication behaviour of tropical fireflies and the idealized behaviour of the flashing patterns. FA uses the following three idealized rules [27–30] to build the mathematical model of the algorithm:

- All fireflies are unisex so that one firefly will be attracted to other fireflies regardless of their sex;
- Attractiveness is proportional to their brightness. Thus for any two flashing fireflies, the less bright one will move towards the brighter one. The attractiveness is proportional to the brightness and they both decrease as their distance increases;
- The brightness of a firefly is affected or determined by the landscape of the objective function. (Thus, for a maximization problem, the brightness can simply be proportional to the value of the objective function.)

In the standard firefly algorithm, there are two important points. One is the formulation of the light intensity and another is the change of the attractiveness. Firstly, we can always assume that the brightness of the firefly can be determined by the encoded objective function landscape. Secondly, we should define the variation of light intensity and formulate the change of the attractiveness. As we know that in nature the light intensity decreases with the distance from its source and the media will absorb the light, so in our simulation we suppose the light intensity *I* varies with the distance *r* and light absorption parameter γ exponentially and monotonically [31]. That is

$$I=I_{0}e^{-\gamma r^{2}}$$ (1)

where *I*_0_ is the original light intensity at the source (i.e., at the distance *r* = 0) and *γ* is the light absorption coefficient. From the idealized rules we known that in our simulation we suppose the attractiveness of firefly is proportional to the light intensity *I*. So we can define the firefly’s light attractive coefficient *β* in the similar way as the light intensity coefficient *I*. That is

$$\beta =\beta_{0}e^{-\gamma r^{2}}$$ (2)

where *β*_0_ is the original light attractiveness at *r* = 0.

The Cartesian distance is used to calculate the distance between any two fireflies *i* and *j* at *x*_*i*_ and *x*_*j*_

$$r_{ij}={\left\|x_{i}-x_{j}\right\|}_{2}=\sqrt{\sum_{k=1}^{d}{\left(x_{i,k}-x_{j,k}\right)}^{2}}$$ (3)

where *d* is the number of dimensions. The amount of movement of firefly *i* to another more attractive (brighter) firefly *j* is determined by

$$x_{i}=x_{i}+\beta_{0}e^{-\gamma r^{2}}(x_{j}-x_{i})+\alpha \varepsilon_{i}$$ (4)

where the first term is the current location of firefly *i*, the second term is due to the attraction, while the third term is randomization with the vector of random variables *ε*_*i*_ being drawn from different distributions such as the Uniform distribution, Gaussian distribution and Lévy flight. In the third term, α is a scaling parameter that controls the step size and it should be linked with the interests of the problems.

According to above idealization and approximations rules, the pseudo-code of standard firefly algorithm can be summarized in Algorithm 1.

**Algorithm 1** Pseudo-code for the standard FA algorithm

Objective function *f*(*x*), *x* = (*x*_1_,⋯,*x*_*D*_)^*T*^

Initialize a population of fireflies *x*_*i*_ (*i* = 1,2,⋯*n*)

Calculate the light intensity *I*_*i*_ at *x*_*i*_ by *f*(*x*_*i*_)

Define light absorption coefficient *γ*

**While (*t* < MaxGeneration**)

        f**or *i* = 1:*n* all *n* fireflies**

                **for *j* = 1:*n* all *n* fireflies**

                    Calculate the distance *r* between *x*_*i*_ and *x*_*j*_ using Cartesian distance equation

                        **if (*I*_*j*_ > *I*_*i*_)**

                            Attractiveness varies with distance *r* via $\beta_{0}e^{-\gamma r^{2}}$

                            Move firefly *i* towards *j* in all *d* dimensions

                            **end if**

                            Evaluate new solutions and update light intensity

                        **end for *j***

        **end for *i***

        Rank the fireflies and find the current best

**end while**

Post-process results and visualization

### Standard Differential Evolution

Differential evolution (DE) was proposed by Storn and Price in 1996, which uses a vectorized mutation operator and two forms of crossover (either exponential or binomial) to evolve from the randomly generated, initial starting points to the potentially optimal solution. There are many DE variants. In this paper, we use the so-called DE*/rand/1/bin* scheme/variant. This variant is probably the most widely used in practice, which can be briefly described as follows [32].

For a given *D*-dimensional minimization problem, a population consists of *n* individual solution vectors. The mutant vector *v*_*i*_ can be defined as follows:

$$v_{i,g+1}=x_{r_{1},g}+F(x_{r_{2},g}-x_{r_{3},g}),\;r_{1}\neq r_{2}\neq r_{3}\neq i$$ (5)

where the indexes *r*_1_,*r*_2_, *r*_3_ ∈ [1, *n*] correspond to three solutions randomly chosen from the whole population and *g* is the iteration/generation index. The indices have to be different from each other. In addition, *F* (*F* ∈ [0,2]) is a perturbation parameter that controls the amplification of the difference vector $x_{r_{2},g}-x_{r_{3},g}$, though in most cases 0 < *F* < 1 is used in practice.

The binomial crossover operation tries to produce a new trial vector from the perturbed or mutated vector *v*_*i*,*g*+1_ = [*v*_*i*1,*g*+1_,*v*_*i*2,*g*+1_,⋯,*v*_*iD*,*g*+1_] and the target vector *x*_*i*,*g*_ = [*x*_*i*1,*g*_,*x*_*i*2,*g*_,⋯,*x*_*iD*,*g*_]

$$u_{i,g+1}=\left\{ \begin{array}{c} v_{ij,g+1,}\\ x_{ij,g,} \end{array}\begin{array}{c} if\;r(j)\leq C_{r}\;or\;j=random(i)\\ if\;r(j)>C_{r}\;or\;j\neq random(i) \end{array}\right.$$ (6)

where *j* ∈ [1,2,⋯*D*], *r*(*j*) is the *jth* realization of a uniform random generator number. In addition, *C*_*r*_ ∈ [0,1] is the so-called crossover constant. Here, *random* ∈ [1,2,⋯,*D*] is a random permutation index vector, which can usually ensure that the trial vector *u*_*i*,*g*+1_ gets at least one character from the mutated vector *v*_*i*,*g*+1_.

The selection mechanism is similar to those of other algorithms where a greedy acceptance is performed:

$$x_{i,g+1}=\left\{ \begin{array}{c} u_{i,g+1},\\ x_{i,g}, \end{array}\begin{array}{c} if\;f(u_{i,g+1})\leq f(x_{i,g})\\ otherwise. \end{array}\right.$$ (7)

This means that the update is accepted only if a better objective is achieved.

Algorithm 2 summarizes the basic steps of the standard differential evolution algorithm.

**Algorithm 2** Pseudo code for the standard DE algorithm

Initialize the population *x*_*i*_ (*i* = 1,2,⋯*n*) from the randomly initial starting points

Set the perturbation parameter *F* and crossover probability parameter *C*_*r*_

**While (*t* < MaxGeneration**)

        f**or *i* = 1:*n* in all individuals**

        For each *x*_*i*_, randomly choose 3 different vectors $x_{r_{1}}$, $x_{r_{2}}$ and $x_{r_{3}}$ from the whole population

        Use mutation to generate a new vector *v*_*i*_

        Generate a random index *random* (*i*)

        Generate a randomly distributed number *r*(*j*) *ϵ* [0,1]

                **for *j* = 1:*D***

                Crossover operation, for each parameter *v*_*ij*_, update

        $u_{i,g+1}=\left\{ \begin{array}{c} v_{ij,g+1,}\\ x_{ij,g,} \end{array}\begin{array}{c} if\;r(j)\leq C_{r}\;or\;j=random(i)\\ if\;r(j)>C_{r}\;or\;j\neq random(i) \end{array}\right.$

                **end for *j***

                Select operation, select and update the solution *x*_*i*_

        **end for *i***

**end** **while**

Post-process results and visualization

## The HFA Algorithm

Both the firefly algorithm and differential evolution have their own advantages and they both work well for a wide range of optimization problems. In this paper, we propose a new hybrid algorithm based on FA and DE by combining some of the advantages of both algorithms. We call the proposed approach the hybrid firefly algorithm (HFA) that combines the attraction mechanism of FA with the mixing ability of DE so as to increase the speed of convergence and the diversity of the population. The major difference between firefly algorithm and differential evolution is how new individuals are generated and then used at each iteration.

Among the many components of algorithms, intensification and diversification (also called exploitation and exploration) are the two major components of any meta-heuristic algorithm [33]. In order to explore the search space on a global scale, meta-heuristic algorithms need to generate a diverse range of solutions using diversification or exploration strategy. Intensification or exploitation strategy can guide the individual to search in a local region, based on the prior knowledge or the new information found during the search process that a current good solution is found in this region. An algorithm’s solution accuracy and convergence rate can be enhanced by balancing intensification and diversification properly.

Firstly, the earlier observations and studies in the literature indicated that the firefly algorithm can subdivide the whole population into subgroups automatically in terms of the attraction mechanism via the variation of light intensity and one of the FA variants can escape from the local minima owing to long-distance mobility by Lévy flight [34]. Such advantages mean that FA is good at exploration as well as diversification. Furthermore, technically speaking, due to the efficiency of mutation operator and crossover operator, differential evolution can provide a good mixing ability among the population and thus provide a better diversity in the population. At the same time, DE can also carry out local search during the process, especially when approaching to the local optimal solutions, and thus we can use this advantage to improve both the exploitation and exploration ability of our proposed algorithm. In addition, updating the current global best in the whole population ensures that solutions can converge to the optimum, while diversification via mixing and regrouping the whole population allows the search algorithm to escape from local optima and may simultaneously increase the diversity of solutions. It is worth pointing out that we only mix and regroup the individual location information obtained after the main iteration of parallel FA and DE processes, rather than generating the new positions from random walks or other operators. The main superiority of such mixing and regrouping mechanism is to guarantee the search focusing on the current locations in the promising areas obtained in the earlier phase instead of having to search or re-search less promising regions of the search space.

Based on above descriptions, the fundamental steps of the HFA can be summarized as the pseudo-code shown in Algorithm 3 where we can see that the parallel use of FA and DE can strike a good balance between exploration and exploitation during the whole iteration process.

**Algorithm 3** Pseudo-code for the HFA algorithm

**Begin**

        Divide the whole group into two groups: *G*_1_ and *G*_2_

        Initialize the populations *G*_1_ and *G*_2_

        Evaluate the fitness value of each particle

        **Repeat**

                **Do in parallel**

                    Perform FA operation on *G*_1_

                    Perform DE operation on *G*_2_

                **End Do in parallel**

                Update the global best in the whole population

                    Mix the two groups and regroup them randomly into new groups: *G*_1_ and *G*_2_

                    Evaluate the fitness value of each particle

        **Until a terminate-condition is met**

**End**

Post-process results and visualization

Though the detailed computational complexity may depend on the structure of the implementation, however, for three meta-heuristic algorithms used in this paper, their complexities can be easily estimated. For FA, the time complexity is O*(n*^2^*t*) where *n* is the population size and *t* is the number of iterations because there are two loops for going through the population. For DE, its complexity is O(*nt*). Therefore, in this case, for our proposed hybrid approach (HFA), the time complexity is O(*n*^2^*t*/4 + *nt*/2) because each component (either FA or DE) only uses half of the population. As n is small (in this case, *n* = 20 *or* 40), and *t* is large (in this case, *t* = 2000), the computation cost is relatively inexpensive because the algorithm complexity is linear in terms of *t*. The main computational cost will be in the evaluations of objective functions.

## Benchmarks and Parameter Settings

### Benchmark Functions

Benchmark functions are useful to evaluate new algorithms and their features such as the precision, the rate of convergence, the robustness and the general performance. To evaluate the performance of our proposed algorithm and other existing algorithms, a set of 13 standard benchmark functions is used and such benchmarks have been chosen with a diverse range of properties. Theoretically speaking, if a small number of the benchmark functions are used, the experimental results may be potential biased due to the limited diversity of the problem objective landscape and in this case it would be very difficult to draw any convincing conclusions. Therefore, we have chosen test functions based on the characteristics, modality and other properties so as to provide a fairly rich set of functions with varied difficulties. In essence, we used the same test functions as those used in [35, 36]. All of the benchmark functions are summarized in Tables 1 and 2 where *D* denotes the dimension of the benchmark function, *S* denotes the scales of the variables, and *F*_*min*_ is the global optimum value in the variable scales.

**Table 1:** Unimodal Benchmark Functions.

Function Name	Function	*D*	*S*	*F*_*min*_
Sphere	$f_{1}\left(x\right)=\sum_{i=1}^{D}x_{i}^{2}$	30	[−100,100]^*D*^	0
Schwefel’s 2.22	$f_{2}\left(x\right)=\sum_{i=1}^{D}\left|x_{i}\right|+\prod_{i=1}^{D}\left|x_{i}\right|$	30	[−10,10]^*D*^	0
Schwefel’s 1.20	$f_{3}\left(x\right)=\sum_{i=1}^{D}{\left(\sum_{j=1}^{D}x_{j}\right)}^{2}$	30	[−100,100]^*D*^	0
Schwefel’s 2.21	*f*_4_(*x*) *= max*_*i*_{|*x*_*i*_|,1 ≤ *i* ≤ *D*}	30	[−100,100]^*D*^	0
Rosenbrock	$f_{5}\left(x\right)=\sum_{i=1}^{D-1}\left[100{\left(x_{i+1}-x_{i}^{2}\right)}^{2}+{\left(x_{i}-1\right)}^{2}\right]$	30	[−30,30]^*D*^	0
Step	$f_{6}\left(x\right)=\sum_{i=1}^{D}{\left(x_{i}+0.5\right)}^{2}$	30	[−100,100]^*D*^	0
Quartic Noise	$f_{7}\left(x\right)=\sum_{i=1}^{n}ix_{i}^{4}+random\left[0,1\right)$	30	[−1.28,1.28]^*D*^	0

**Table 2:** Multimodal Benchmark Functions.

Function Name	Function	D	S	*F*_*min*_
Schwefel’s 2.26	$f_{8}\left(x\right)=\sum_{i=1}^{D}-x_{i}\sin\left(\sqrt{\left|x_{i}\right|}\right)$	30	[−500,500]^*D*^	-12569.5
Rastrigin	$f_{9}\left(x\right)=\sum_{i=1}^{D}\left[x_{i}^{2}-10\cos\left(2\pi x_{i}+10\right)\right]$	30	[−5.12,5.12]^*D*^	0
Ackley	$\begin{array}{l} f_{10}\left(X\right)=-20\exp\left(-0.2\sqrt{\frac{1}{n}\sum_{i=1}^{n}x_{i}^{2}}\right)\\ -\exp\left(\frac{1}{n}\sum_{i=1}^{n}\cos\left(2\pi x_{i}\right)\right)+20+e \end{array}$	30	[−32,32]^*D*^	0
Griewank	$f_{11}\left(X\right)=\frac{1}{4000}\sum_{i=1}^{n}x_{i}^{2}-\prod_{i=1}^{n}\cos\left(\frac{x_{i}}{\sqrt{i}}\right)+1$	30	[−600,600]^*D*^	0
Pendlized	$\begin{array}{l} f_{12}\left(x\right)=\sum_{i=1}^{D}u\left(x_{i},10,100,4\right)\\ +\frac{\pi }{D}\left\{10\sin^{2}\left(3\pi y_{i}\right)+\sum_{i=1}^{D-1}{\left(y_{i}-1\right)}^{2}\left[1+\sin^{2}\left(3\pi y_{i+1}\right)\right]+{\left(y_{D}-1\right)}^{2}\right\} \end{array}$ $y_{i}=1+\frac{1}{4}\left(x_{i}+1\right)$ $u\left(x_{i},a,k,m\right)=\{\begin{array}{c} k{\left(x_{i}-1\right)}^{m},\\ 0,\\ k{\left(-x_{i}-1\right)}^{m}, \end{array}\begin{array}{c} x_{i}>a,\\ -a\leq x_{i}\leq a,\\ x_{i}<-a, \end{array}$	30	[−50,50]^*D*^	0
Generalized Pendlized	$\begin{array}{l} f_{13}\left(x\right)=\sum_{i=1}^{D}u\left(x_{i},5,10,4\right)\\ +\frac{1}{10}\left\{\sin^{2}\left(3\pi x_{1}\right)+\sum_{i=1}^{D-1}{\left(x_{i}-1\right)}^{2}\left[1+\sin^{2}\left(3\pi x_{i+1}\right)\right]+{\left(x_{D}-1\right)}^{2}\left[1+\sin^{2}\left(2\pi x_{D}\right)\right]\right\} \end{array}$ $u\left(x_{i},a,k,m\right)=\{\begin{array}{c} k{\left(x_{i}-1\right)}^{m},\\ 0,\\ k{\left(-x_{i}-1\right)}^{m}, \end{array}\begin{array}{c} x_{i}>a,\\ -a\leq x_{i}\leq a,\\ x_{i}<-a, \end{array}$	30	[−50,50]^*D*^	0

The test benchmark functions can be divided into two groups in terms of the number of local minima: unimodal functions and multimodal functions. The unimodal test functions have one global optimum, so they are suitable for benchmarking the local exploitation ability of algorithms. This kind of functions will allow to focus more on the convergence rates of the tested algorithms other than the final results. Multimodal test functions have many local minima, and the number of local optima usually increases exponentially with the problem dimension, so they are suitable for benchmarking the global exploration ability of algorithms. This kind of multimodal functions can test the exploration ability which can make the algorithm escape from local optima. In some applications, to find a good optimal or suboptimal solution is more important, while other applications may place the emphasis on the accuracy of the solutions. So the quality of final results is more of concern in such applications.

From Table 1, we know that functions *f*_1_-*f*_7_ are unimodal, high-dimensional problems. Function *f*_5_, also namely the ‘banana function’, has a global optimum inside a long but flat, narrow, parabola-shaped valley. To find the location of the valley is non-trivial, though not too difficult. However, to converge to the global minimum with a high accuracy is more difficult, especially for gradient-based algorithms. Function *f*_6_ is the step function, characterized by plateaus and discontinuities. In addition, function *f*_7_ is a noisy quadratic function.

Functions *f*_8_–*f*_13_ in Table 2 are multimodal, high-dimensional problems and more details are summarized in Table 2. For example, *f*_8_ is a non-convex, multimodal and additively separable function. This seemingly simple function can be deceptive because the global minimum at (420.9687,⋯,420.9687) is geometrically distant from the next best local minima in the domain [−500,500]^*D*^ where *D* is the number of dimensions. Therefore, many algorithms including some of metaheuristic algorithms may find it quite challenging to solve. In addition, *f*_9_ is also challenging as it is one of the most difficult benchmarks commonly used in the literature because it has multiple, steep wells with multiple local minima. Another widely used multimodal benchmark function is *f*_10_, namely the Ackley function, which can be characterized by a deep valley at the centre and an almost flat outer zone. Consequently, it is quite challenging to solve because it is easy for most optimization algorithms to get trapped in one of its many local minima due to the multimodality.

### Parameter Settings

For the verification purpose of the algorithms and the analysis of the experimental results, our proposed hybrid firefly algorithm is compared to the standard FA and DE as well as PSO to benchmark the performance and to see if there is any improvement.

In all cases, the population size is set to 40, and the dimension of the benchmark functions is equal to 30. We also set the maximum number of iterations, as the stopping criteria, equal to 2000. The initial population is generated using uniformly distributed random initialization within the ranges or limits of the design variables. In addition, 30 independent runs have also been carried out for each function and each algorithm with completely different initial settings. The results from the algorithms are accompanied according to four standard statistical measures: the Minimum, the Maximum, the Mean, and the Standard Deviation (Std) of the fitness values calculated over 30 independent runs.

For the firefly algorithm, we set the initial attractiveness *β*_0_ = 2 * *rand*, the light absorption coefficient *γ* = 1/*S*^2^ where *S* donates the average range of the variables, the random parameter *α* (*α* = 0.2 * 0.95^*iter*^ where 0.2 is the initial randomness factor and *iter* is the index of the iteration) reduces monotonically and gradually. Finally, we use the Lévy distribution to draw the random numbers because it can produce occasionally some long leaps [37]. The values of the differential evolution algorithm-dependent parameters are *F* = 0.5 as the scaling factor and *C*_*r*_ = 0.9 as the crossover constant [38]. Additionally, for particle swarm optimization, the learning factors *c*_1_ and *c*_2_ are both set as 2, the inertia weight *ω* decreases linearly from *ω*_*max*_ = 0.9 to *ω*_*min*_ = 0.4 [39].

It is worth pointing out that in our proposed HFA, the parameters, *β*_0_, *γ*, *α*, *ε*_*i*_, *F* and *CR*, are all the same as those defined in the standard FA and DE. Specially, in our implementations, we have divided the whole population into two subgroups (subpopulations), which means that the population size in FA and DE each is equal to 20. And at the same time we have also divided the total 2000 iterations into 10 sub-iteration groups (or subgroups or substages). For each sub-iteration group, FA and DE, respectively, the number of sub-iterations is set to 200 times in parallel, and thus the total of 2000 iterations is realized in 10 subgroups and each with a number of 200 iterations.

All of the algorithm-dependent parameters are summarised in Table 3.

**Table 3:** Algorithm-dependent parameters of the comparison algorithms.

Algorithm	Control parameters			
FA	*β*_0_ = 2 * *rand*	*γ* = 1/*S*^2^	*α* = 0.2 * 0.95^*iter*^	*ε*_*i*_ = *L*é*vy flight*
DE	*F* = 0.5		*C*_*r*_ = 0.9	
PSO	*ω*_*max*_ = 0.9	*ω*_*min*_ = 0.4	*c*_1_ = 2	*c*_2_ = 2

### Experimental Results and Analysis

#### Unimodal Function Experimental Results

In the first series of experiments, the aim is to compare the exploitation ability and convergence rate of the mentioned algorithms for functions *f*_1_-*f*_7_. The statistic results of 30 independent runs are given in Table 4. The best mean results of the algorithms are written in bold.

**Table 4:** Results of unimodal benchmark functions.

# *Fnc*	Statistics	HFA	FA	DE	PSO
*f*_1_	Min	1.07E-193	1.02E-87	1.3949e-09	6.33E-13
	Max	7.84E-170	1.95E-87	6.1265e-08	6.86E-10
	Mean	**2.64E-171**	1.57E-87	1.4155e-08	9.43E-11
	Std	0	1.89E-88	1.2949e-08	1.48E-10
*f*_2_	Min	1.40E-117	1.56E-44	1.7950e-04	3.21E-09
	Max	7.39E-102	1.95E-44	0.0013	2.48E-07
	Mean	**2.46E-103**	1.73E-44	6.0678e-04	3.66E-08
	Std	1.35E-102	8.52E-46	2.8188e-04	4.69E-08
*f*_3_	Min	1.97E-66	2.3801	0.0454	127.3
	Max	1.30E-55	97.594	1.2297	1237.8
	Mean	**5.30E-57**	25.714	0.2789	459.69
	Std	2.42E-56	24.013	0.2844	241.9
*f*_4_	Min	2.05E-05	1.37E-44	0.3745	2.787
	Max	2.5528	1.86E-44	2.3055	12.205
	Mean	0.7115	**1.68E-44**	0.8832	6.5934
	Std	0.76784	1.30E-45	0.3877	2.2382
*f*_5_	Min	2.47E-29	26.346	14.9121	1.8755
	Max	0.53092	89.131	25.2670	114.49
	Mean	**0.077152**	29.053	21.9994	49.686
	Std	0.16183	11.348	2.1032	34.029
*f*_6_	Min	0	0	0	0
	Max	0	0	0	0
	Mean	**0**	**0**	**0**	**0**
	Std	0	0	0	0
*f*_7_	Min	7.15E-05	0.000518	0.0029	0.013704
	Max	0.000296	0.003894	0.0239	0.04622
	Mean	**0.000183**	0.001582	0.0113	0.031475
	Std	5.07E-05	0.000797	0.0045	0.008425

As can be seen from Table 4, HFA performs significantly better than FA, DE and PSO consistently for all unimodal test functions except for *f*_4_. For *f*_4_, our proposed HFA cannot tune itself successfully, whereas FA solves this function quite accurately. In essence, this case is consistent with the so-called no-free-lunch (NFL) theorems. This means that there is no universally superior algorithm for all types of problems [40, 41]. However, as we are not intending to solve all types of problems, therefore, ranking algorithms is always possible for any given set of problems.

In the rest of this section, we use Freidman tests to test which of the mentioned algorithms are statistically better in the solution of benchmark functions [42]. A null hypothesis indicates that two algorithms are equivalent and, therefore, they can get the equal ranks. If the performance of the algorithms is statistically different, the null hypothesis will be rejected. We use a significance level 0.95 (or α = 0.05) for the Friedman tests. Table 5 summarises the mean values of all the relevant unimodal benchmark functions. The results of the Friedman non-parametric test are illustrated in Table 6. According to the p-values in Table 6, we can conclude that HFA has a significant difference from FA and PSO. However, the result become insignificant when compared with DE.

**Table 5:** The mean value of unimodal benchmark functions for HFA, FA, DE and PSO over 30 runs.

# *Fnc*	HFA	FA	DE	PSO
*f*_1_	2.64E-171	1.57E-87	1.4155e-08	9.43E-11
*f*_2_	2.46E-103	1.73E-44	6.0678e-04	3.66E-08
*f*_3_	0.7115	25.714	0.2789	459.69
*f*_4_	5.30E-57	1.68E-44	0.8832	6.5934
*f*_5_	0.077152	29.053	21.9994	49.686
*f*_6_	0	0	0	0
*f*_7_	0.000183	0.001582	0.0113	0.031475

**Table 6:** P-values at *α* = 0.05 by Friedman test.

T-test	HFA -FA	HFA -DE	HFA-PSO
P	0.0143	0.1025	0.0143

**Fig 1:**
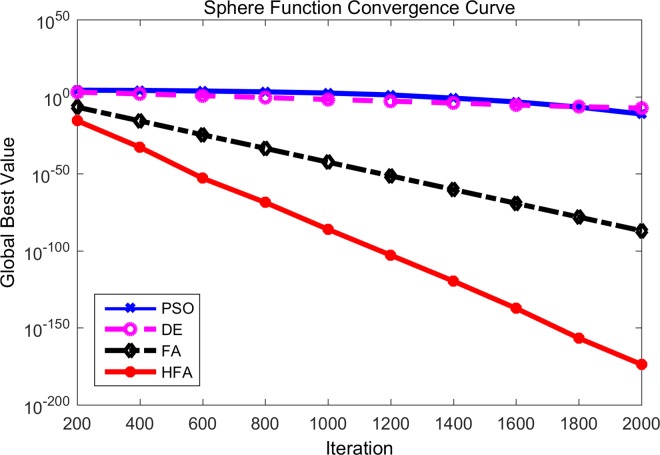
Comparison between PSO, DE, FA and HFA for the Sphere function.

**Fig 2:**
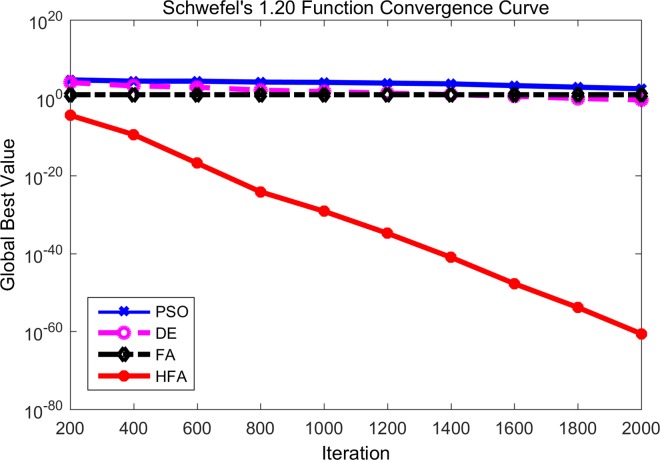
Comparison between PSO, DE, FA and HFA for Schwefel’s 1.20 function.

**Fig 3:**
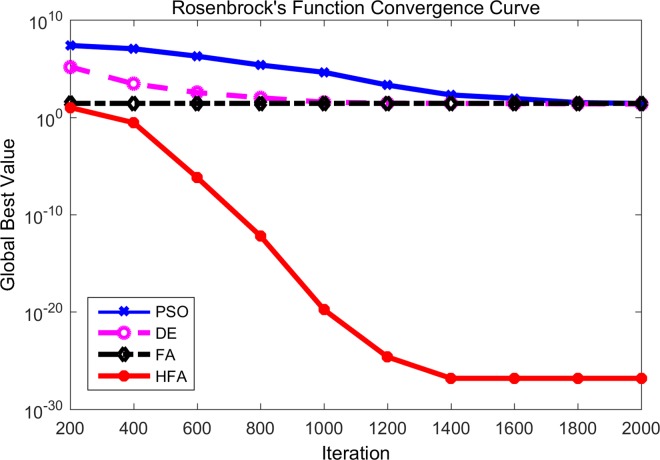
Comparison between PSO, DE, FA and HFA for Rosenbrock’s function.

Figs 1–3 are the convergence curves observed by the 4 mentioned algorithms for *f*_1_, *f*_3_ and *f*_5_. In these figures, the horizontal axis is the number of iterations and the vertical axis is the fitness value of the benchmark function. It is can be seen that HFA performs significantly better than FA, DE, and PSO. For example, *f*_1_, namely the simple sphere function, is a famous benchmark function. During the whole generations, HFA displays a faster convergence rate than those of FA, DE, and PSO due to its better exploitation search ability. It is clear that HFA quickly reaches the neighborhood of the global optimum and gets approximately 10^−16^ with only 200 iterations, while DE and PSO can only reach approximately 10^−12^ and 10^−8^, respectively after the final 2000 iterations. In fact, HFA has a nearly constant convergence rate throughout the whole iteration for most of the unimodal benchmark functions. And the experiment results of HFA after 200 iterations (which means only one iterative repetition time) are better than the final results of DE and PSO after 2000 iterations. Hence from Figs 1–3, we can say that our proposed HFA has a quicker convergence rate and is able to improve its results steadily for a long time. On the other hand, FA also maintains a fast convergence rate at the beginning, however, it can get stuck into the local optimum very soon especially for Figs 2 and 3. Hence, we can know that FA cannot prevent premature convergence due to the poor exploration ability, especially as the iterations proceed. From the observed convergence curves, it is clear that DE and PSO have a very low convergence rate during the whole process compared with HFA and FA.

#### Multimodal Functions

For the second series of experiments, we use multimodal functions to compare the exploration ability of the compared algorithms. The statistical results of comparing the mentioned algorithms with 30 independent runs are presented in Table 7. The best mean results of the mentioned algorithms are written in bold.

**Table 7:** Results of multimodal benchmark functions.

# *Fnc*	Statistics	HFA	FA	DE	PSO
*f*_8_	Min	-12569	-10596	-5627.9	-10001
	Max	-12214	-8424.1	-4515.8	-7093.8
	Mean	**-12439**	-9469.5	-5016.3	-9020.8
	Std	133.24	515.47	260.0659	515.74
*f*_9_	Min	1.08E-08	3.9798	143.8889	17.909
	Max	4.36E-08	15.919	196.3629	44.773
	Mean	**3.39E-08**	9.3858	175.9112	30.15
	Std	7.29E-09	3.0436	12.24334	7.1079
*f*_10_	Min	4.44E-15	7.99E-15	4.3632e-05	4.75E-07
	Max	6.13E-05	1.51E-14	0.0031	6.15E-05
	Mean	1.31E-05	**1.25E-14**	3.1272e-04	7.10E-06
	Std	2.33E-05	3.36E-15	5.4874e-04	1.47E-05
*f*_11_	Min	0	0	1.8299e-07	3.74E-12
	Max	5.64E-08	0	0.1005	0.046483
	Mean	5.86E-09	**0**	0.0132	0.013444
	Std	1.19E-08	0	0.0220	0.012311
*f*_12_	Min	1.57E-32	1.57E-32	1.8989e-09	2.34E-12
	Max	1.57E-32	1.57E-32	0.0036	0.31096
	Mean	**1.57E-32**	**1.57E-32**	2.2768e-04	0.024188
	Std	5.57E-48	5.57E-48	6.8779e-04	0.064897
*f*_13_	Min	1.35E-32	1.35E-32	6.6202e-08	2.42E-11
	Max	1.35E-32	1.35E-32	7.1684e-05	0.010987
	Mean	**1.35E-32**	**1.35E-32**	1.1956e-05	0.0032963
	Std	5.57E-48	5.57E-48	1.7650e-05	0.0051211

From the statistic results in Table 7 we can know that the HFA outperformed other compared algorithms when solving the functions *f*_8_ and *f*_9_. The FA is the best for solving the functions *f*_10_ and *f*_11_. In addition, HFA and FA have almost equal optimization abilities for solving the functions *f*_12_ and *f*_13_. Both can obtain the accurate results of these functions.

Similar to what we have done for the unimodal test functions, the Friedman tests using the significance level of 0.95 (or α = 0.05) are also conducted for all the multimodal benchmark functions. Table 8 summarizes the mean values of the final results over 30 independent runs. The results of these tests are summarized in Table 9. The P-value in Table 9 shows that HFA has a significant difference from DE, while the results become insignificant when compared with FA and PSO.

**Table 8:** The mean value of multimodal benchmark functions for HFA, FA, DE and PSO over 30 runs.

# *Fnc*	HFA	FA	DE	PSO
*f*_8_	-12439	-9469.5	-5016.3	-9020.8
*f*_9_	3.39E-08	9.3858	175.9112	30.15
*f*_10_	1.31E-05	1.25E-14	3.1272e-04	7.10E-06
*f*_11_	5.86E-09	0	0.0132	0.013444
*f*_12_	1.57E-32	1.57E-32	2.2768e-04	0.024188
*f*_13_	1.35E-32	1.35E-32	1.1956e-05	0.0032963

**Table 9:** P-values at *α* = 0.05 by Friedman test.

T-test	HFA -FA	HFA -DE	HFA-PSO
P	1.0000	0.0143	0.1025

**Fig 4:**
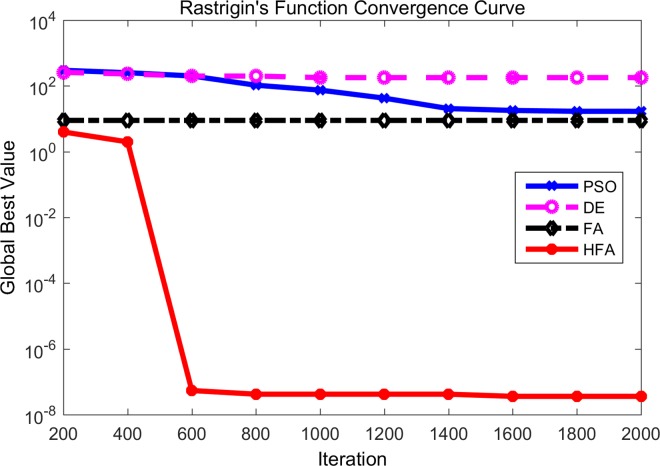
Comparison between PSO, DE, FA and HFA for Rastrigin’s function.

**Fig 5:**
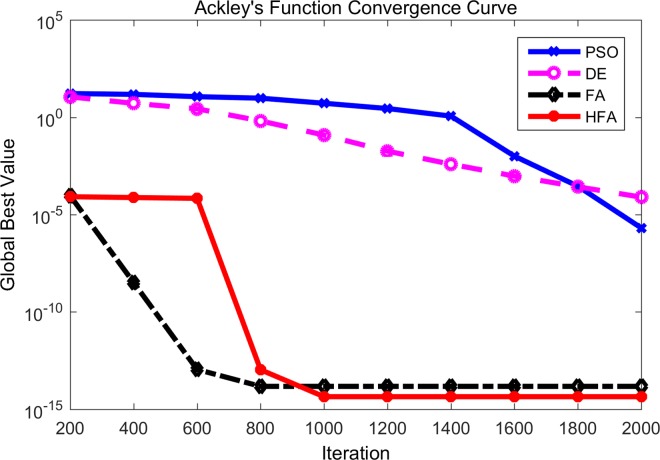
Comparison between PSO, DE, FA and HFA for Ackley’s function.

At the same time, the convergence curves of different algorithms for *f*_9_ and *f*_10_ have been shown in Figs 4 and 5 where the horizontal axis is the number of iterations and the vertical axis is the fitness value of the benchmark function. According to Fig 4, DE and PSO perform poorly during the whole iterative process. FA maintains a higher convergence rate, but unfortunately it appears to become plunged into local optima after about 200 iterations. HFA can escape from the local optima automatically and find the final global best. As can be seen in Fig 5, it is obvious that FA and HFA perform significantly better than DE and PSO. In the beginning, FA displays a faster convergence rate than HFA, while HFA overtakes FA finally. Thus we can say that for the Ackley function both HFA and FA can maintain a strong exploration ability and robustness.

## Conclusions

In this paper, we have proposed a novel hybrid firefly algorithm (HFA) by combining some of the advantages of both firefly algorithm and differential evolution. Based on the theoretical analysis and the problem solving ability of metaheuristic algorithms, we can summarize that HFA has three advantages or improvements: the first strategy is equipped with a better balance between exploration and exploitation due to the parallel use of FA and DE and the population information-sharing. The experimental results illustrated that FA can provide an excellent convergence rate and a strong exploration ability, whereas DE is good at exploitation by using mutation and crossover operators. Ideally, an algorithm should explore the search space as extensively as possible to find all the promising regions and simultaneously it should conduct a more refined search in the promising areas so as to improve the precision of the solutions.

The second improvement is that the selection mechanism used in the proposed approach can enable the solution to converge to the optimum in a better way. This is achieved by first mixing the two subpopulations that are independently evolved using either FA or DE, and then selecting the best solutions among both subpopuations. Thus, it is more likely to find the global optimum than each individual algorithm involved in the hybrid. The third strategy improvement is that the hybrid can increase the diversity of solutions efficiently and can also help the algorithm avoid the stagnation problem by using a mixing and regrouping mechanism. It can be observed that the attraction operator in FA is a double-edged sword. To some extent, it can accelerate the convergence speed, but may also mislead the algorithm to get stuck into some local optima if the diversity of the population becomes low. Technically speaking, this hybrid mechanism can liberate the population from sub-optimal solutions and enable a continued progress toward the true global optima as have been observed in the simulations.

The statistical analyses have also confirmed the theoretical insight in this paper that the three enhancements in the combined approach can explore and exploit the search space more efficiently. It has been seen from the above results that the proposed HFA can indeed work well compared to FA, DE and PSO, which has been further confirmed by the results obtained from the Friedman tests.

Future work will explore different ways of mixing and regrouping the population so as to enhance the performance even further. In addition, it will be useful to carry out a more detailed parametric study to see how different sub-stages of iterations can be used to maximize the parallelism and also to reduce the overall number of iterations. Furthermore, it will also be useful to automatically tune these parameters depending on the modality of the problem and thus can solve problems more effectively in real-world applications.
